# Covariation of scleral remodeling and PI3K/Akt signaling pathway in experimental myopia

**DOI:** 10.1038/s41598-025-97643-7

**Published:** 2025-04-11

**Authors:** Xiaomeng Li, Xiaojing Liu, Yingxin Yu, Tuling Li, Lijie Guo, Guili Hu, Huixia Wei, Zhaohui Yang, Jinpeng Liu, Yixian Hao, Ruixue Zhang, Qiuxin Wu, Xuan Liao, Dadong Guo, Hongsheng Bi

**Affiliations:** 1 Shandong University of Traditional Chinese Medicine, Jinan, 250014 China; 2 School of Ophthalmology and Optometry, Wenzhou Medical University, Wenzhou, 325027 China; 3 Guangzhou laboratory, Guangzhou Medical University, Guangzhou, 510000 China; 4 Affiliated Eye Hospital of Shandong University of Traditional Chinese Medicine, No. 48#, Yingxiongshan Road, Jinan, 250002 Shandong China; 5 Department of Ophthalmology of Affiliated Hospital and Medical School of Ophthalmology and Optometry, North Sichuan Medical College, Nanchong, 637000 China; 6 Shandong Provincial Key Laboratory of Integrated Traditional Chinese and Western Medicine for Prevention and Therapy of Ocular Diseases, Jinan, 250002 China; 7 Shandong Academy of Eye Disease Prevention and Therapy, Jinan, 250002 China; 8 Medical College of Optometry and Ophthalmology, Shandong University of Traditional Chinese Medicine, Jinan, 250002 China; 9 Medical College of Optometry and Ophthalmology, Shandong Academy of Eye Disease Prevention and Therapy, Shandong University of Traditional Chinese Medicine, No. 48#, Yingxiongshan Road, Jinan, 250002 Shandong China

**Keywords:** Experimental myopia, PI3K/Akt signaling pathway, TGF-β1, Collagen, Scleral remodeling, Genetics, Molecular biology, Medical research, Molecular medicine

## Abstract

The present study aimed to investigate the role of the PI3K/Akt signaling pathway in scleral remodeling in the development of negative lens-induced myopia (LIM). The change of scleral morphology in experimental myopic guinea pigs was observed by transmission electron microscopy, Masson staining, and TUNEL assay, respectively. Meanwhile, the levels of the PI3K/AKT signaling pathway- and scleral remodeling-related molecules in scleral tissues were determined by real-time quantitative PCR (qPCR), enzyme-linked immunosorbent assay (ELISA), immunofluorescence, immunohistochemical staining, and western blot, respectively. We found that 2-week myopic induction can elevate PIK3R3 and AKT2 levels and activate the PI3K/Akt signaling pathway, enhance the expression of E-cadherin and matrix metallopeptidase 2 (MMP2), and decrease the level of transforming growth factor-beta 1 (TGF-β1), tissue inhibitor of matrix metalloproteinase-2 (TIMP2), and collagen (COLI) in the scleral tissue of myopic guinea pigs, thereby leading to scleral remolding. However, 4-week myopic induction could inhibit the PI3K/AKT signaling pathway and induce apoptosis, accompanied by increased MMP2, E-cadherin, and decreased TGF-β1, TIMP2, and COLI. Results reveal that the disturbed PI3K/AKT signaling plays a role in scleral remodeling in the experimental myopia through orchestrating apoptosis.

**Supplementary Information:**

The online version contains supplementary material available at 10.1038/s41598-025-97643-7.

## Introduction

Myopia is one of the most common eye diseases in children and adolescents worldwide, leading to a series of complications, including cataracts, myopic macular degeneration, retinal detachment, and open-angle glaucoma. It is reported that the prevalence of myopia is the highest in East and Southeast Asia in young people ranging from 80 to 90% and 10–20% for high myopia^1^.

Any degree of myopia may increase the risk of adverse changes in ocular tissues. Following the epidemiologic investigation, one-third of high myopic patients have the risk of bilateral low vision with the growth of age; however, the risk of complications in low and moderate myopia is already high^2^. Presently, the number of myopic patients is increasing year by year, and the age of myopic patients is young. It is predicted that by the middle of the 21st century, people suffering from myopia will be up to half of the world’s population^3^. Currently, there is no specific and effective method for the treatment of myopia, and the pathogenesis of myopia is still unclear. Researchers have found that several signaling pathways, including TGF-β, Wnt/β-catenin, and IGF-1 signaling pathways, are associated with the process of myopia^4–6^. In addition, Li reported that PI3K/AKT/mTOR signaling participates in the insulin-mediated regulation of pathological myopia-related factors in retinal pigment epithelial cells^7^. Nevertheless, the detailed mechanism of myopia needs to be further explored.

The sclera is a typical connective tissue whose biomechanical qualities are determined by the content and distribution of biopolymers^8^. The size and shape of the eye are determined by this connective tissue, which is mainly the fibrous collagen scaffold in the hydrated interfibrillar matrix composed of proteoglycans and glycoproteins^9^. A large number of studies have shown that scleral remodeling is a key event in the development of myopia. During the development of myopia, the axial length grows continuously, and the sclera is strained and deformed, leading to scleral remodeling. In addition, loss of scleral tissue involves decreased connective tissue synthesis and increased degradation of collagen typeI (COLI), which ultimately leads to adverse changes in the composition of the sclera and its ductility^10,11^. The visual stimulation that produces myopia will reduce the synthesis of scleral proteoglycans, increase the decomposition of scleral collagens^12^, and the synthesis of enzymes that degrade the scleral extracellular matrix (ECM)^13^. It will lead to alterations in the structural components of the ECM, make the sclera thinner, change its biomechanical properties, and make it easier to increase the axial elongation^14^.

An increasing number of studies have shown that the phosphatidylinositol 3 kinase/protein kinase B (PI3K/Akt) signaling pathway is abnormally activated in a variety of ophthalmic diseases and regulates the occurrence and development of ophthalmopathy. It is closely related to cell migration, differentiation, and apoptosis as well as protein synthesis^15^. PI3K is a dimer composed of a catalytic subunit and a regulatory subunit named phosphoinositide-3-kinase regulatory subunit 3 (PIK3R3). When it binds to the receptor, PIK3R3 can change the protein structure of Akt and activate it, thereby activating or inhibiting a series of downstream substrates by AKT phosphorylation. Ando et al. analyzed miRNAs in the vitreous of macular hole patients undergoing vitrectomy and found that, compared to patients without high myopia, patients with high myopia expressed lower levels of miRNAs that targeted 32 signaling pathways, including the PI3K/AKT pathway^16^. Li et al. indicated that in retinal pigment epithelial cells, insulin regulated the secretion of pathological myopia-related factors through the PI3K/AKT/mTOR signaling pathway and thus promoted the development of pathological myopia via transducing regulatory signals from retina to choroid and sclera^7^. These results indicate that the PI3K/AKT signaling pathway plays a part in the development of myopia.

To investigate the significance of disturbed PI3K/Akt signaling and related molecules in the induction of lens-induced myopia in guinea pigs, in the present study, we established a negative lens-induced myopia (LIM) guinea pig model. Further, we investigated the changes of PIK3R3, AKT2 and TGF-β1, E-cadherin, COLI, BCL-2, BAD, MMP2, and TIMP2 in the sclera of myopic guinea pigs to explore their effects on the development of myopia after 2- and 4-week myopic induction. Our results gain new insights into the role of PI3K/AKT signaling in bringing about LIM scleral remodeling, providing ideas for the establishment of a new potential target in treating myopia.

## Materials and methods

### Animals

This study is performed in accordance with relevant guidelines and regulations. All methods are reported in accordance with ARRIVE guidelines. This study was approved by the Experimental Animal Ethics Review Committee of Affiliated Hospital of Shandong University of Traditional Chinese Medicine (Approval number: AWE-2022-055). The use and feeding of experimental animals were strictly performed in accordance with the ARVO Statement for the Use of Animals in Ophthalmic and Vision Research. In the present study, 120 healthy British tricolor short-haired guinea pigs (male, 110–130 g, 2 weeks old, Jinan Jinfeng Biotechnology Co. Ltd., Jinan, China) were used to perform the relevant experiments. The animals were randomly divided into two main groups: NC group (*n* = 60) and LIM group (*n* = 60). Each main group was further divided into two subgroups based on the duration of the experiment: 2-week NC (*n* = 30), 2-week LIM (*n* = 30), 4-week NC (*n* = 30), and 4-week LIM (*n* = 30) subgrops. Among them, 76 guinea pigs were utilized for specific experimental analyses, including transmission electron microscopy (TEM) (*n* = 6), immunofluorescence (*n* = 6), Masson staining (*n* = 6), immunohistochemical staining (*n* = 6), TUNEL assay (*n* = 6), Ingenuity Pathway Analysis (*n* = 6), qPCR (*n* = 12), Western Blot (*n* = 16), and ELISA (*n* = 12). The remaining 44 guinea pigs were reserved as backups to ensure the robustness and reliability of the experimental procedures. Husbandry conditions were as follows: the vivarium was maintained at 22–25{degree sign}C with white ambient lighting at an average of 500 lx on a 12 h-on/12 h-off schedule, and animals were fed standard guinea pig lab chow ad libitum. After excluding cataracts, corneal disease, spontaneous myopia, and other eye diseases, animals were randomly divided into two groups: a normal control (NC) group and a lens-induced myopia (LIM) group. The NC and LIM groups were further divided into 2-week NC, 2-week LIM, 4-week NC, and 4-week LIM subgroups. Animals in each group were raised either for 2 weeks or 4 weeks under the same conditions. Among them, the eyes of guinea pigs in the NC group were left untreated, while the LIM group comprises of animals with lens-induced unilateral myopization by goggles with a refractive power of − 6.00D glued onto the orbital rim of right eyes and were left untreated in the left eyes. The animals were checked twice a day in the morning and evening to clean the dirty lens with swab or to refix lens in front of the eye if needed.

### Biometric measurements

The lenses were gently removed prior to lens-treatment and after 2 and 4 weeks of myopia induction, then 10 mg/mL of cyclopentolate hydrochloride eye drops (Alcon, USA) were administered to the conjunctival sac of the guinea pigs three times to dilate the pupils, with an interval of 5 min. After the pupils were completely dilated, the dioptric test was performed by a streak retinoscope (YZ24, 66 VISION TECH, China) and the working distance was 50 cm. The final value of the diopter was the average of the two main meridian tests, vertical and horizontal. Each eye was measured at least 6 times, and the average of the stable values was regarded as the experimental result. All refractive parameters were detected by the same optometrist in a dark room.

Ophthalmic A-scan ultrasound (Cinescan; Quantel medical, Cournon-d’auvergne, France) was used to measure the axial length, anterior chamber depth, lens thickness, and vitreous chamber depth of guinea pigs. Instrument parameter settings were set as follows: the anterior chamber propagation velocity was 1557 m/s, the lens propagation velocity was 1723 m/s, and the vitreous propagation velocity was 1540 m/s. Prior to measurement, 2 drops of topical anesthetic (obuprocaine hydrochloride, Mito Pharmaceutical Co., Osaka, Japan) were applied to the cornea of each eye. The average of 10 repeated measurements that fell within an acceptable error range of [standard deviation ≤ 0.05] was taken as the experimental result. All measurements were done by the same optometrist.

### Transmission electron microscopy

Three guinea pigs were randomly chosen from each group after myopia induction for 4 weeks. In the beginning, 10 g/L of sodium pentobarbital was injected intraperitoneally at a body weight of 8 ml/Kg, and then the right eyeballs were taken out and immediately fixed in a mixed solution containing 2% paraformaldehyde (PFA) and 2.5% glutaraldehyde. After fixation for 2 h, 1 mm cryostat sections were cut from the posterior segment of the eye, which includes sclera, choroid, and retina, fixed in 2.5% glutaraldehyde solution, and washed with 0.1 M PBS (10 min×3), fixed with 1% osmic acid for 1 h, washed with 0.1 M PBS (pH = 7.2), dehydrated with different gradients of alcohol (e.g., 50%, 70%, 90%, and 100%), and then the samples were embedded in resin (SPI-PON 812 RESIN, America) and cut into semi-thin slices. The sections were stained with 3% uranyl acetate and lead citrate, and finally observed with TEM (Hitachi HT7800, Japan).

### Immunofluorescence assay

After 4-week myopia induction, 3 guinea pigs in each group were euthanized by intraperitoneal injection of 10 g/L sodium pentobarbital. The eyes of the guinea pigs were extracted and washed in cold sterile physiological saline immediately, and then the tissues around the eyes were removed. The eyes were fixed in eye fixation solution (Servicebio, Wuhan, China) for 24 h, and were subjected to routine dehydration, paraffin embedding, and sectioning. Tissue sections were placed in EDTA antigen retrieval buffer (pH 8.0) for antigen repair and washed with PBS (5 min×3), BSA was added dropwise and blocked for 30 min. Next, primary antibodies against TGF-β1 (1:1000, BIOSS, Beijing, China) and E-cadherin (1:1000, BIOSS, Beijing, China) were added and incubated overnight at 4 °C. Further, the slides were rinsed with PBS, followed by incubation with the secondary antibody (Servicebio, Wuhan, China) for 50 min at room temperature in a dark room. At the indicated time, the slides were washed with PBS and incubated with 4’6-diamidino-2-phenylindole (DAPI) dye solution at room temperature for 10 min in a dark room. To quench the spontaneous tissue fluorescence, the slides were rinsed with PBS, quenched with an autofluorescence (Servicebio, Wuhan, China) quenching agent for 5 min, and rinsed with running water for 10 min. After the slides were slightly dried, the slides were sealed with an anti-fluorescence quenching sealing agent and observed with a fluorescence microscope (Nikon Eclipse, 55i, Japan).

### Masson staining

The procedure for making paraffin sections was the same as above. After the sections were dewaxed, they were successively immersed in Masson A solution, Masson B and Masson C mixed solution, 1% hydrochloric acid alcohol, and Masson D solution (Servicebio, Wuhan, China). After each immersion, the slides were rinsed with tap water and then dipped into the Masson E solution. Subsequently, they were slightly drained and directly dipped into the Masson F solution. After that, they were rinsed and differentiated with 1% glacial acetic acid and dehydrated with two cylinders of absolute ethanol. Soak the sections in anhydrous ethanol and xylene for 5 min each, and then seal them with neutral gum. After sealing, the fluorescent microscope (Nikon eclipse C1, Nikon, Japan) was used for microscopic examination.

### Immunohistochemical staining

After 4-week myopia induction, three guinea pigs in each group were anesthetized by intraperitoneal injection of 10 g/L sodium pentobarbital, then the eyes of the guinea pigs were extracted and washed in cold sterile saline immediately, followed by the removal of the tissues around the eyes. Further, the eyes were fixed in an eye fixation solution (G1109, Servicebio, Wuhan, China) for 24 h and were subjected to routine dehydration, paraffin embedding, and sectioning. After rinsing with PBS (pH7.2), each section was placed in a 3% H_2_O_2_ solution and incubated at room temperature for 25 min in the darkness. After rinsing with PBS, 3% BSA (GC305010, Servicebio, Wuhan, China) was added to the sections and incubated at room temperature for 30 min. After rinsing with PBS, anti-COLIprimary antibody (1:1000, ABclonal Technology Co., Ltd., Wuhan, China) was incubated overnight at 4 °C. Further, the slices were rinsed with PBS and incubated with HRP-labeled secondary antibody at room temperature for 50 min, and then rinsed with PBS twice, followed by incubation with DAB (G1212, Servicebio, China) for 5 min. Finally, the slices were observed under a white light microscope (E100, Nikon, Japan). Quantification of COLI levels in the NC and LIM groups was assessed using ImageJ software (National Institutes of Health, USA) based on DAB staining color intensity.

### TUNEL assay

We randomly selected 3 guinea pigs (4-week myopia induction) in each group to isolate the sclera to perform a TUNEL assay. Briefly, the guinea pigs were euthanized and the sclera tissues of the guinea pigs in each group (*n* = 3) were isolated, then paraffin sections of the sclera were dewaxed, repaired with proteinase K working solution, incubated in an incubator at 37 °C for 22 min, and then washed with PBS. The tissue was covered with the membrane-breaking working solution (Servicebio, Wuhan, China) and incubated for 20 min at room temperature. After washing with PBS, the tissue was incubated for 10 min in PBS buffer at room temperature. The TUNEL reaction solution from the TUNEL assay kit (Servicebio, Wuhan, China) was added to the wet box after slicing and incubated at 37 °C for 2 h. After washing with PBS, the DAPI dye solution was added dropwise and incubated at room temperature for 10 min. After sufficient washing with PBS, the slides were sealed with an anti-fluorescence quenching sealing agent, and then conducted microscopic observation under a fluorescent microscope (Nikon, Eclipse, C1, Tokyo, Japan). The scleral nuclei stained by DAPI showed blue, and the positive apoptotic nuclei displayed by FITC showed green. To identify the rate of apoptotic cells in each group, two independent researchers calculated cells from more than 5 random microscopic fields.

### RNA-Seq and ingenuity pathway analysis

To investigate the molecular mechanisms involved in scleral remodeling in myopic guinea pigs, we performed RNA sequencing (RNA-Seq) on scleral tissues. After 2-week myopia induction, the sclera tissues from three guinea pigs in each group were analyzed for KEGG pathway enrichment and GO functional annotation. Based on the enriched pathways and functional annotations, we selected appropriate pathways and related genes for detection. We chose molecules associated with biological functions and/or diseases from the QIAGEN knowledge base, applying fold change thresholds ≥ 1.5 or ≤ 0.67, combined with *P* < 0.05. The right-tailed Fisher’s exact test was employed to calculate P-values, determining the probability that the observed associations between biological functions/diseases and the dataset occurred by chance alone. Finally, differentially expressed genes (DEGs) were analyzed using QIAGEN Ingenuity Pathway Analysis (IPA) software (http://www.qiagen.com/ingenuity) to construct regulatory networks and annotate functional pathways.

### Real-time quantitative PCR

After inducing myopia for 2 and 4 weeks, 3 guinea pigs were randomly selected from each group for euthanasia and their right eyeballs was removed. Subsequently, cut along the corneoscleral margin to remove anterior segment tissues such as cornea, iris, and lens, peel off the vitreous body, remove the retina and choroid, and obtain posterior scleral tissue. Total RNA was extracted from scleral tissues of 4 subgroups (*n* = 3) of guinea pigs using an improved SparkEasy tissue/cellular RNA rapid extraction kit (Shandong Sparkjade Science Co. Ltd., Jinan, China), and the purity and concentration of RNA were measured by an ultraviolet spectrophotometer (K5600, Beijing Kaiao Technology Development Co. Ltd., Beijing, China), followed by the synthesis of cDNA using a 1st Strand cDNA Synthesis Kit (Shandong Sparkjade Science Co. Ltd., Jinan, China) in 200µL enzyme-free PCR tubes (NEST Biotech., Wuxi, China). For qPCR determination, primer sequences were designed by the DNAStar software and synthesized by Shanghai Sangong Bioengineering Co., Ltd. The qPCR amplification was performed using the ChamQ Universal SYBR qPCR Master Mix kit (Vazyme Biotech Co., Ltd., Nanjing, China) in 96-well PCR plates (NEST Biotech, Wuxi, China). The qPCR conditions were as follows: 94 °C for 5 s, 1 cycle; 94 °C for 5 s, 54 °C for 15 s, and 72 °C for 10 s, 45 cycles. The result was assessed using the 2^-△△Ct^ method^17^. The primer sequences are listed in Table 1.

**Table 1:** The primer sequences of target genes detected by qPCR.

Gene	Primer sequence
PIK3R3	F: 5’-GTTGCCGATGGGGAAGTG-3’ R: 5’-GTGCGTGGACAGGGTAGG-3’
AKT2	F: 5’-CAGAGGATCGGGCACGGTTTTACG-3’ R: 5’-GGCGGCCGCACATCATCTCATACA-3’
TGF-β1	F: 5’-AACCGGCCCTTCCTGCTCCTCAT-3’ R: 5’-CGCCGGGGTTGTGCTGGTTGTA-3’
E-cadherin	F:5’-ACGGCCCCTGCAGTTTCACAA-3’ R:5’-AACGGGCCTTTTTCATTTTCTG-3’
MMP2	F: 5’-TTTTTCCCCGCAAGCCCAAGTG-3’ R: 5’-TCAAAGTGCGAGTCTCCCCCAACC-3’
TIMP2	F: 5’-AAGGCGGTCAGTGAGAAGGAGGTA-3’ R: 5’-CCGAGGAAGGGGCTGTGTAGATAA-3’
BCL-2	F: 5’-GCACCCCCGGCATCTTCTCCTTCC-3’ R: 5’-GGCGTCCCATCCTCCGTTATCCTG-3’
BAD	F: 5’-GGAGGCACTGTGGCTATGGAGACC-3’ R: 5’-AGCGGCCCCGGAATGGACTGAGC-3’
COLI	F: 5’-GCTGGTGCAAGTGGTGGTGGTTAT-3’ R: 5’-CATGTGCGAGCGGGGTTCTTC-3’
GAPDH	F:5’-CTGACCTGCCGCCTGGAGAAACC-3’ R:5’-ATGCCAGCCCCAGCGTCAAAAGT-3’

Abbreviations: qPCR, quantitative real-time PCR; RT, reverse transcription primer; F, forward primer; R, reverse primer.

### Western blot

After 2 and 4 weeks of myopia induction, scleral tissues from 4 guinea pigs in each group were isolated. The samples were ground under liquid nitrogen, added RIPA lysis buffer containing PMSF according to the mass volume ratio (10 mg:100µL), and then sonicated on ice; the supernatants were obtained after centrifugation at 6000 g by using a centrifuge (NEST Biotech., Wuxi, China) for 10 min at 4 °C. The scleral protein concentration of each sample was measured by using a BCA protein concentration determination kit (Beyond Biotechnology Co. Ltd., Shanghai, China), then proteins were separated by a 10% separation gel electrophoresis kit (Shandong Sparkjade Science Co. Ltd., Jinan, China), followed by transfer on polyvinylidene fluoride (PVDF) membrane. Blots were blocked with 5% bovine serum albumin (BSA) for 1 h. Further, the PVDF membrane was incubated with the corresponding primary antibody anti-PIK3R3 (1:500, Boster, Wuhan, China), anti-phosphorylated (p)-AKT2 (1:500, Cusabio, Wuhan, China), anti-TGF-β1 (1:1000, YaJi Biological, Shanghai, China), anti-E-Cadherin (1:1000, BIOSS, Beijing, China), anti-BAD (1:1000, BIOSS, Beijing, China), anti-BCL-2 (1:4000, BIOSS, Beijing, China), anti-COLI(1:1000, ABclonal Technology Co., Ltd., Wuhan, China), and anti-GAPDH (1:10000, Proteintech, Wuhan, China) at 4 °C overnight. After rinsing with TBST, Goat anti-rabbit IgG antibody labeled with horseradish peroxidase (1:10000, Sparkjade Science Co. Ltd., Jinan, China) was incubated at 4 °C for 1 h. Finally, the Fusion-FX7 imaging system (Vilber lourmat, Marne La Vall ée, France) was used for development. GAPDH protein was used as the loading reference, and quantitative analysis was performed using Image J 7.0 software (National Institutes of Health, USA).

### Enzyme-linked immunosorbent assay

After 2- and 4-week myopia induction, guinea pigs were euthanized with an overdose of 10 g/L of sodium pentobarbital, then the scleral tissues from three guinea pigs of each group were isolated from the eyes. Proteins were isolated and quantified as above for western blots. The levels of MMP2 and TIMP2 were detected with the relevant ELISA Kits (Jianglai Biotechnology Co. Ltd., Shanghai, China).

### Statistical analysis

GraphPad Prism 9.0 software was used for statistical analysis. In this study, the data are presented in the form of mean ± standard deviation (SD). A paired t test was done for each refractive parameter between the eyes at different time points, and an independent sample t-test was done for the right eye of experimental myopia and the ipsilateral eye of the normal control animals. An independent sample t-test was carried out for the NC and LIM groups of gene and target protein expression. *P* < 0.05 was considered statistically significant.

## Results

### Changes in refraction and axial length

**Fig. 1:**
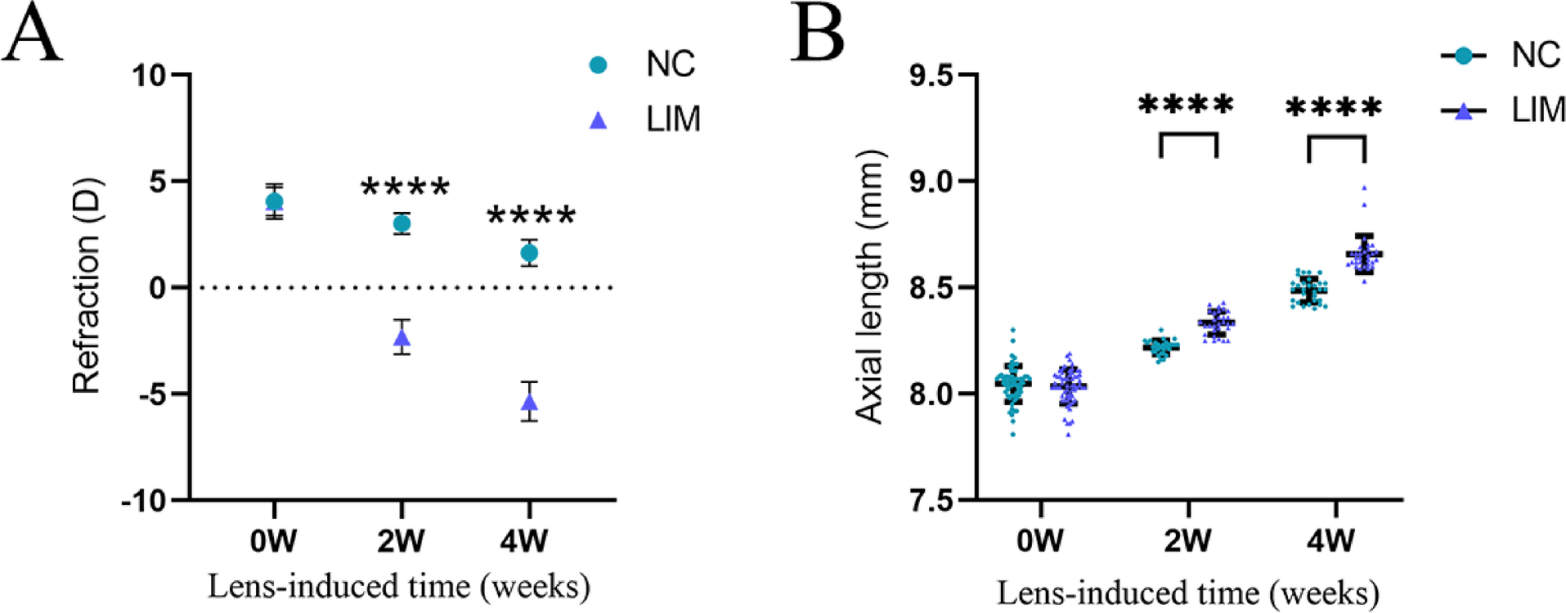
Comparison of refraction (**A**) and axial length (**B**) of the right eyes of guinea pigs in the NC and LIM groups after 2- and 4-week myopic induction. Significance refers to comparisons of the LIM group to the NC group (*** *p* < 0.001, **** *p* < 0.0001). *N* = 30.

After 2 and 4 weeks of myopia induction, the refraction of the experimental myopia eye in the LIM group increased compared with that of the ipsilateral eye of guinea pigs in the NC group, and the decrease of refraction was more obvious with the extension of myopia induction (Fig. 1A, ****P < 0.001). The axial length of the experimental myopia eye in the LIM group also increased with the extension of myopia induction compared with that of the ipsilateral eye of guinea pigs in the NC group (Fig. 1B, ****P < 0.0001). This result indicates that negative lens induction in guinea pigs can increase the diopter of the eye and axial length, leading to experimental myopia.

### Transmission electron microscopy

**Fig. 2:**
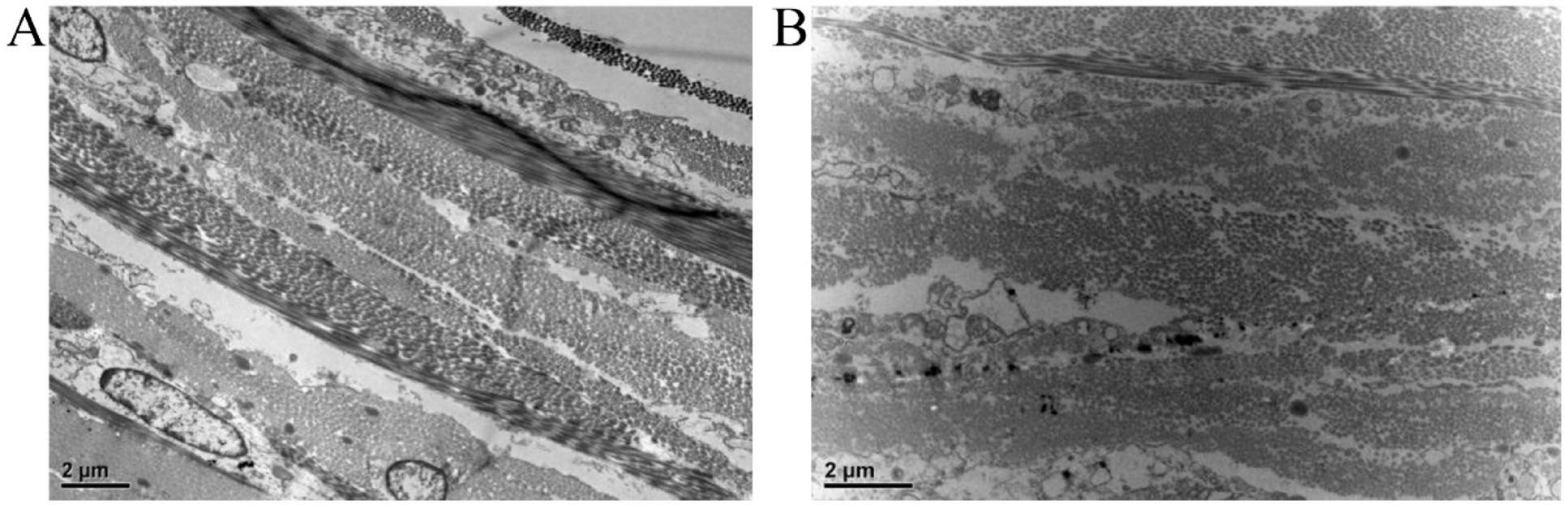
Morphological changes of sclera tissues of NC (**A**) and LIM (**B**) guinea pigs. After 4-week myopia induction, the scleral tissues of the right eyes in the LIM group were disorganized, and the arrangements of collagen fibrils became loose compared with those of the NC group. Bar = 2 μm. *N* = 3.

After 4-week myopia induction, the TEM analysis showed that the sclera tissue of guinea pigs in the NC group had a regular structure, and collagen fibrils were arranged tightly (Fig. 2A). In contrast, the scleral tissue structure of guinea pigs in the LIM group was disorganized, and collagen fibrils were thinner with loose arrangements (Fig. 2B). The reduction and abnormal arrangement of these collagen fibers reflect the pathological characteristics of scleral remodeling, suggesting an imbalance in the metabolism of the ECM of the sclera after myopia induction, which leads to a decrease in its biomechanical strength and further exacerbates the irreversible changes in ocular axial length and refractive power.

### TGF-β1 and E-cadherin expression detected by Immunofluorescence assay

**Fig. 3:**
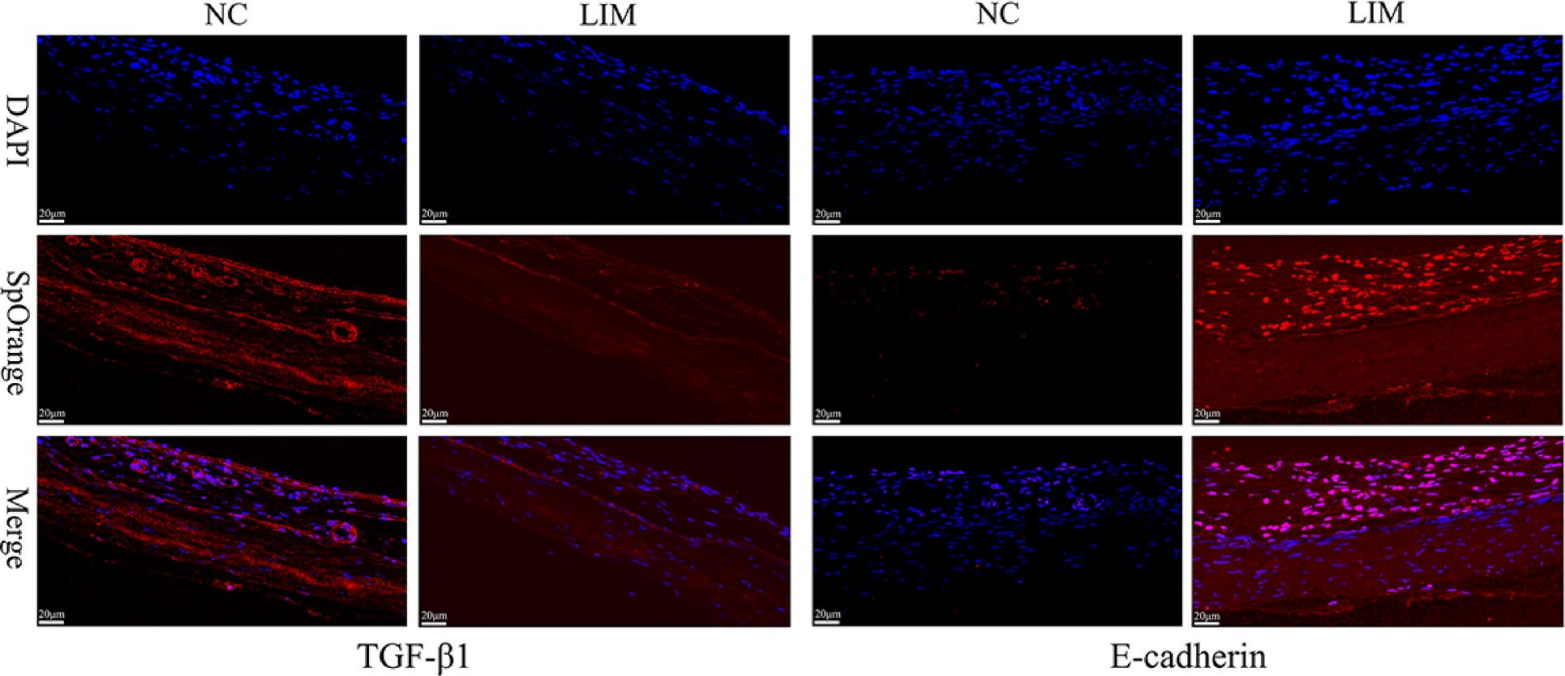
Comparison of TGF-β1 and E-cadherin expression in sclera in the NC and LIM groups. The samples were detected by immunofluorescent staining after 4-week myopia induction. The results showed that DAPI staining presented blue, and the target protein presented red. Magnification = 40×. Bar = 20 μm. *N* = 3.

To determine the levels of TGF-β1 and E-cadherin in the sclera of myopic guinea pigs, we performed immunofluorescence staining (Fig. 3). The results showed that after 4 weeks of myopia induction, the expression of TGF-β1 in scleral tissues of guinea pigs in the LIM group was lower than that in the NC group. However, E-cadherin expression in scleral tissues in the LIM group was stronger than that of the NC group.

### Masson staining

**Fig. 4:**
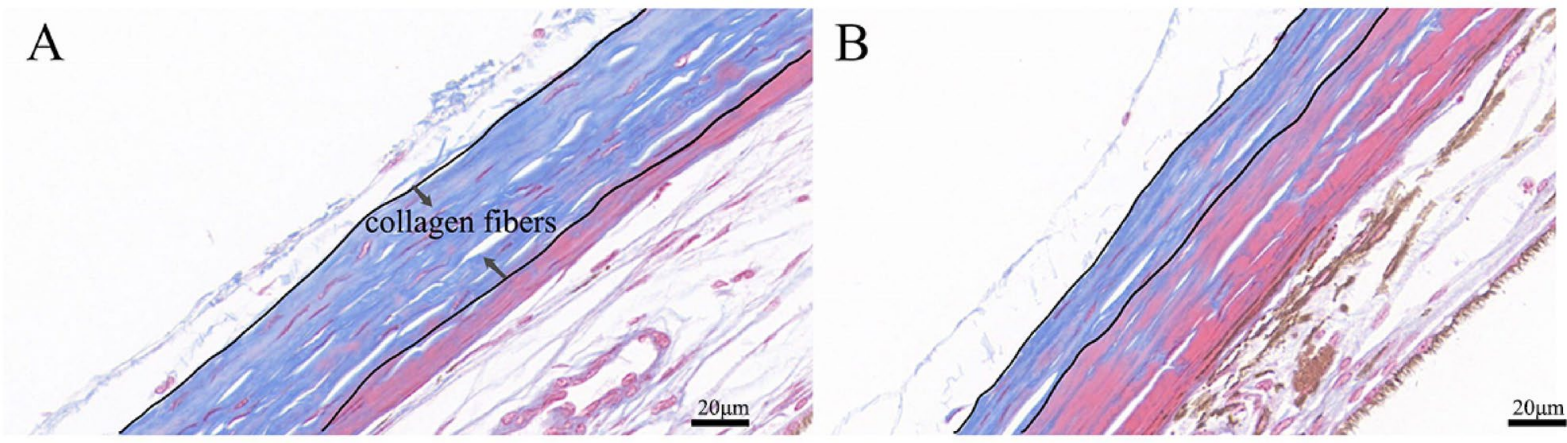
Comparison of sclera tissue of guinea pigs in the NC (A) and LIM (B) groups after 4-week myopia induction based on Masson staining. Compared with the NC group, the collagen fibers (blue) in the LIM group reduced, whereas the muscle fiber cellulose (red) increased. Magnification = 40×. Bar = 20 μm. *N* = 3.

To explore the effect of 4-week myopia induction on the changes in the sclera, we performed Masson staining. Collagen fibers appeared blue, and muscle fiber cellulose appeared red. Results indicated that the level of scleral collagen fibers in the LIM group (Fig. 4B) was reduced compared with that of the NC group (Fig. 4A).

### COLI expression detected by immunohistochemical assay

**Fig. 5:**
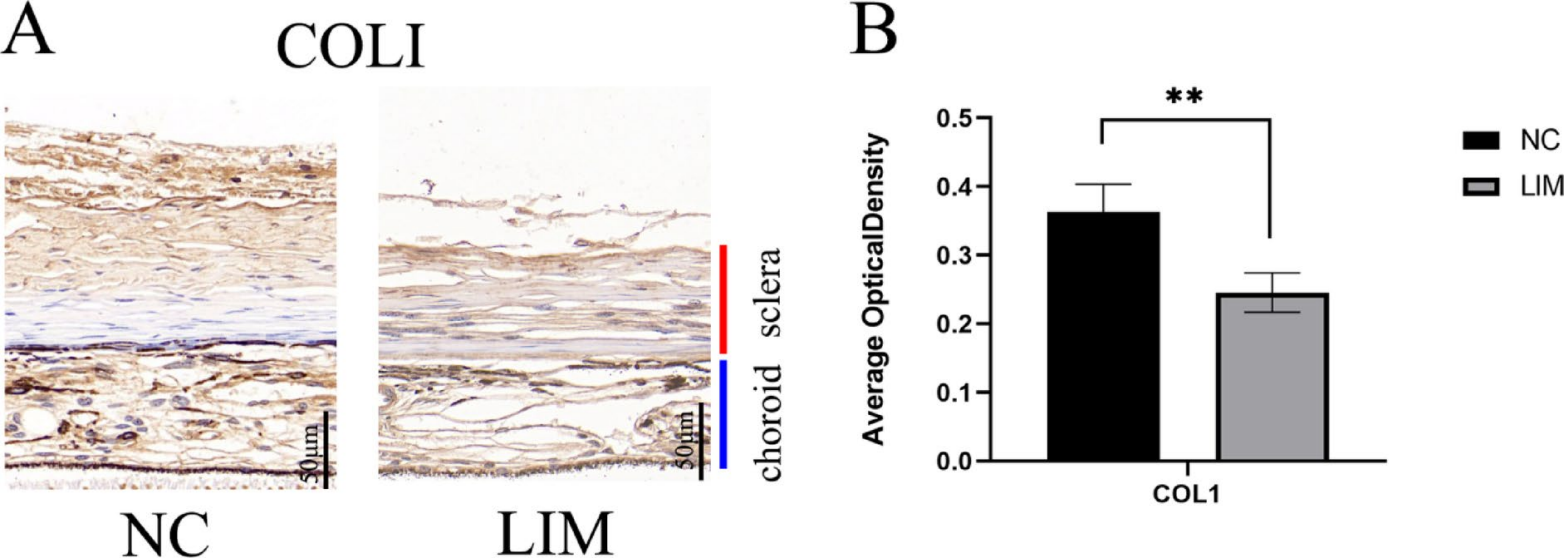
Immunohistochemical assay of the sclera tissue of guinea pigs in the NC and LIM groups after 4-week myopia induction. After DAB staining, the collagen fibers were stained brownish yellow (**A**). Quantification of COLI levels in the NC and LIM groups was assessed using ImageJ based on DAB staining color intensity (**B**). Data presented as mean ± SD (**p < 0.01). Magnification = 20×. Bar = 50 μm. *N* = 3.

As shown in Fig. 5, we noted that after 4-week myopia induction, the expression of COLI in the sclera in the LIM group was significantly lower than the level of the NC group, indicating that COLI was closely correlated with scleral remodeling, and the reduction of collagen caused the persistence of scleral remodeling (***P < 0.001).

### TUNEL assay

**Fig. 6:**
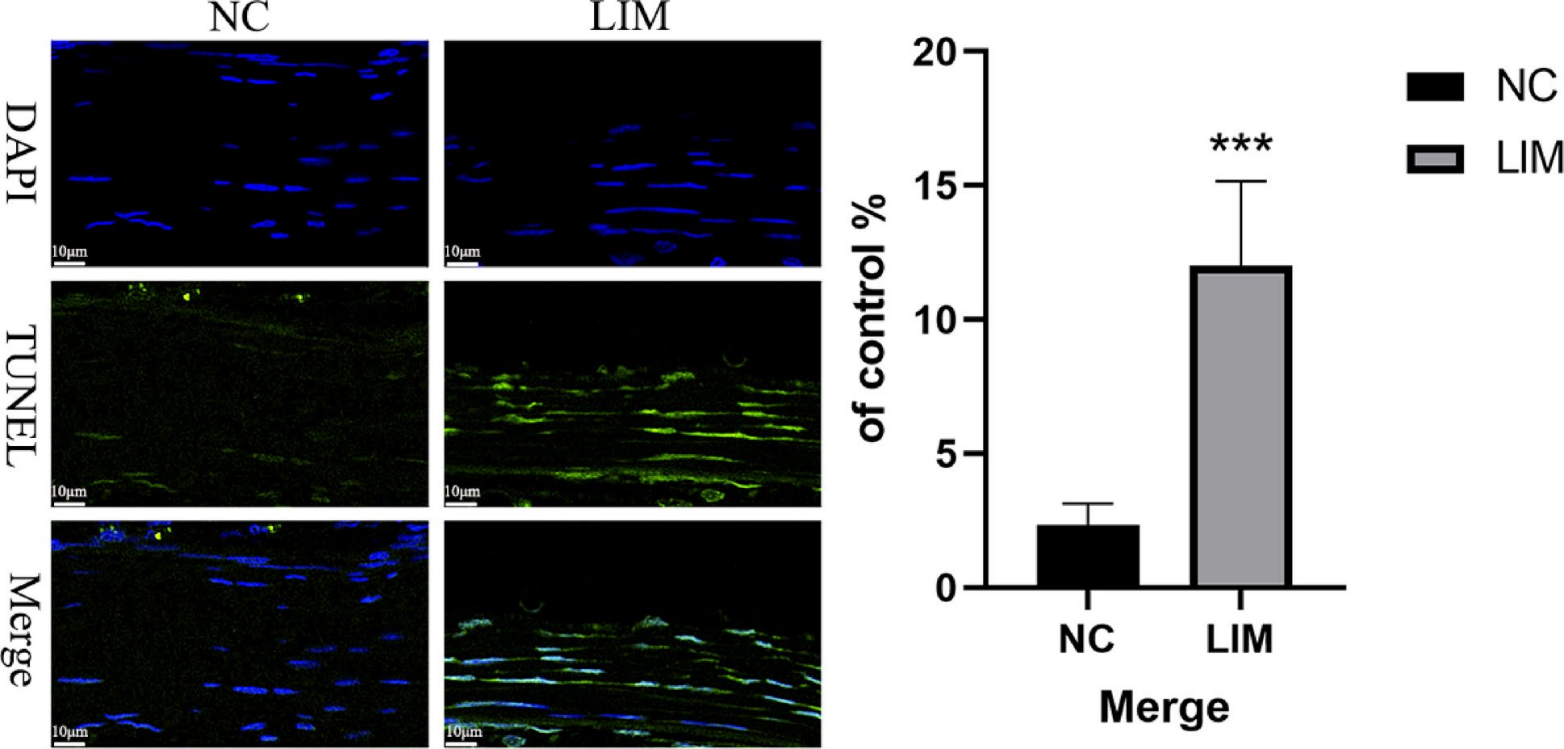
Comparison of the apoptotic cells of the sclera in NC and LIM groups detected by TUNEL staining after 4-week myopia induction. Results indicated that the scleral nuclei stained by DAPI showed blue, and the positive apoptotic nuclei displayed by FITC showed green. Significance refers to comparisons of the LIM group to the NC group (*** *p* < 0.001). Magnification: 80×. Bar = 10 μm. *N* = 3.

To investigate the effect of myopia induction on apoptosis of scleral cells in guinea pigs after 4-week myopia induction, we performed TUNEL staining. As shown in Fig. 6, we observed an increase in the number of TUNEL-positive cells in the sclera in the LIM group compared with the number of the NC group.

### Ingenuity pathway analysis

**Fig. 7:**
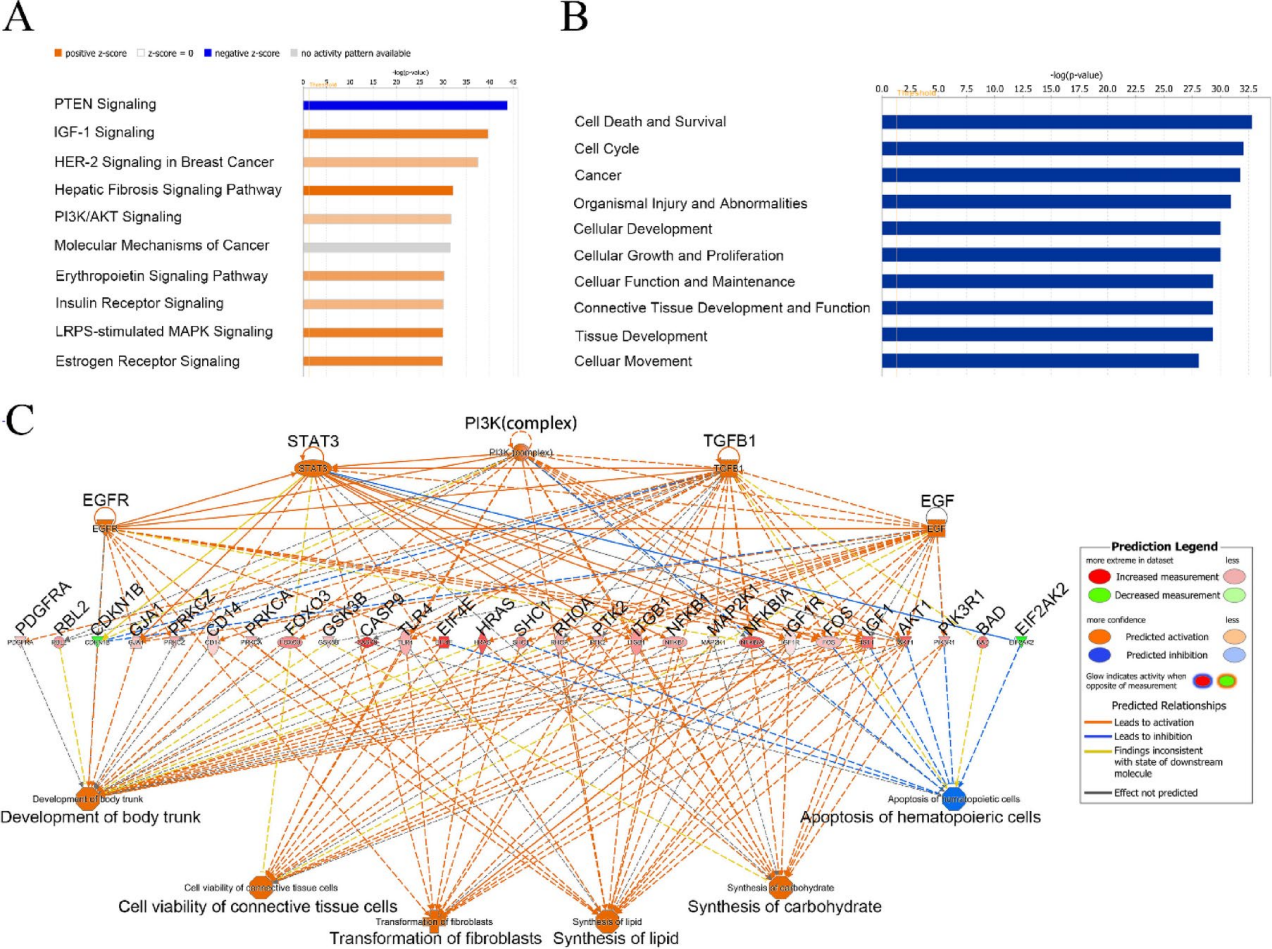
Bioinformatics annotation analyzed by RNA-seq and IPA. The differentially expressed genes related to PI3K/AKT signaling pathway was used to perform the KEGG pathway analysis, and the top 10 significantly enriched signaling pathways (ranked by enrichment significance) was demonstrated (A), the Gene Ontology (GO) functional annotation analysis of the RNA-seq dataset (B), and the regulatory effect analysis (C). The solid lines indicate the direct interaction between the two genes, while the dotted line indicates the indirect relationship. The length of the line reflects the strength of the reported evidence supporting the relationship between nodes, and the shape of the nodes represents the different known biological roles of these molecules. *N* = 3.

After RNA-seq, we selected the differentially expressed genes related to PI3K/AKT signaling pathway to perform the KEGG pathway analysis. We found that the top 10 enriched typical paths with negative logarithm (P value) and found that IGF-1 and PI3K/Akt signaling pathways were significantly activated, whereas the PTEN signaling was inhibited in the sclera in experimental myopic guinea pigs (Fig. 7A). Meanwhile, the Gene Ontology (GO) functional annotation analysis showed that differentially expressed genes (such as PIK3R3 and AKT2) could play a role in the occurrence and development of myopia by affecting cell survival and function as well as cell migration (Fig. 7B). In addition, based on IPA analysis, the regulatory effect analysis showed the possible pathways of the upstream regulatory network and downstream molecular function between the genes, in which the PI3K/AKT and TGF-β signaling pathways were correlated with the development of myopia, mainly involving apoptosis, cell viability, and transformation of fibroblasts (Fig. 7C).

### qPCR analysis

**Fig. 8:**
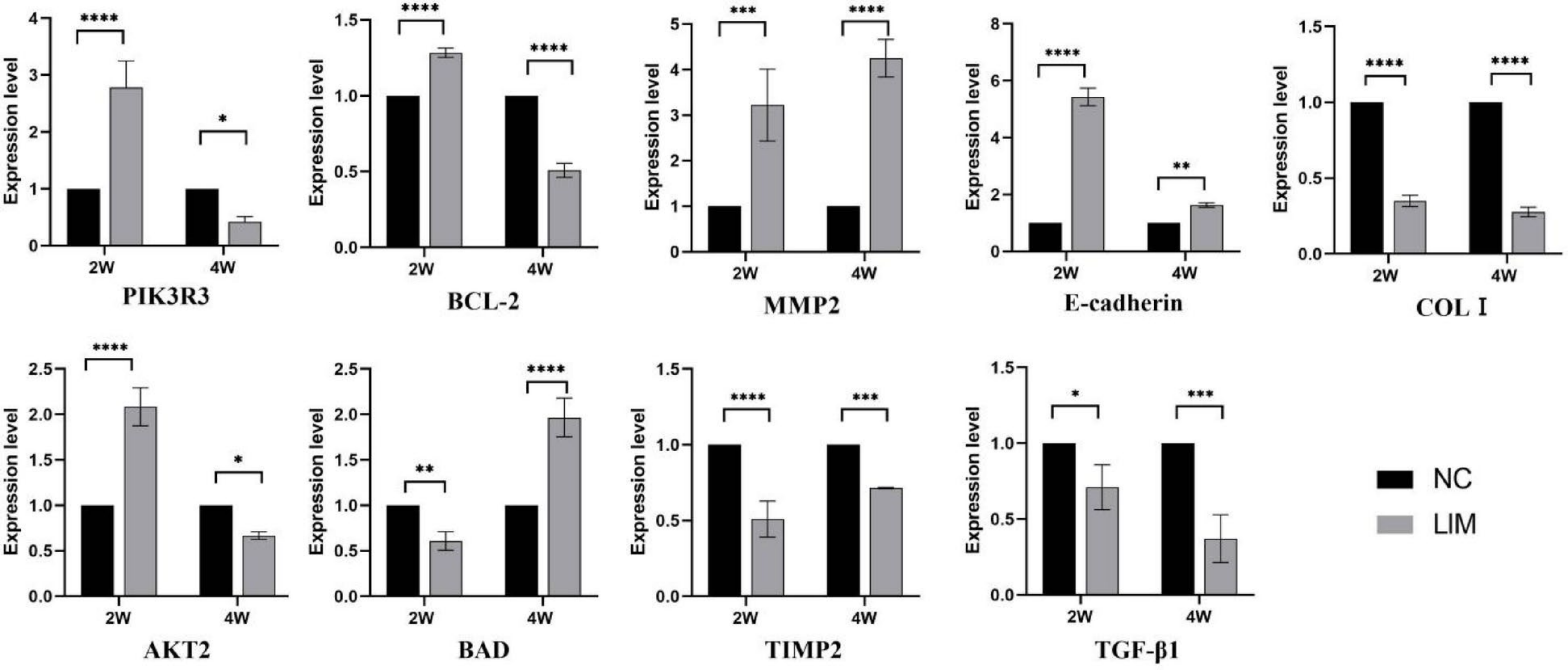
Determination of the relative gene expression. The levels of PIK3R3, AKT2, TGF-β1, E-cadherin, MMP2, TIMP2, BAD, BCL-2, and COLI were detected by qPCR after myopia induction. Significance refers to comparisons of the LIM group to the NC group (* *p* < 0.05, ** *p* < 0.01, *** *p* < 0.001, **** *p* < 0.0001). *N* = 3.

After myopia induction for 2 and 4 weeks, the expression of PIK3R3, AKT2, BAD, BCL-2, TGF-β1, E-cadherin, COLI, MMP2, and TIMP2 was detected by qPCR. As shown in Fig. 8, the results indicated that the gene expression of PIK3R3, AKT2, BCL-2, E-cadherin, and MMP2 in the sclera of experimental myopic guinea pigs was significantly higher than the levels of the control animals after 2 weeks of myopia induction, whereas the mRNA expression of BAD, TGF-β1, COLI, and TIMP2 was decreased; in contrast, after 4-week myopia induction, PIK3R3, AKT2, and BCL-2 were decreased, whereas BAD was up-regulated. At the same time, the levels of TGF-β1, COLI, and TIMP2 genes were still significantly decreased compared with the level of the NC group, whereas the E-cadherin and MMP2 mRNA levels were significantly increased (*P < 0.05, **P < 0.01, ***P < 0.001, and ****P < 0.0001).

### PIK3R3, p-AKT2, BAD, BCL-2, E-cadherin, TGF-β1, and COLI expression in scleral tissues detected by western blot

**Fig. 9:**
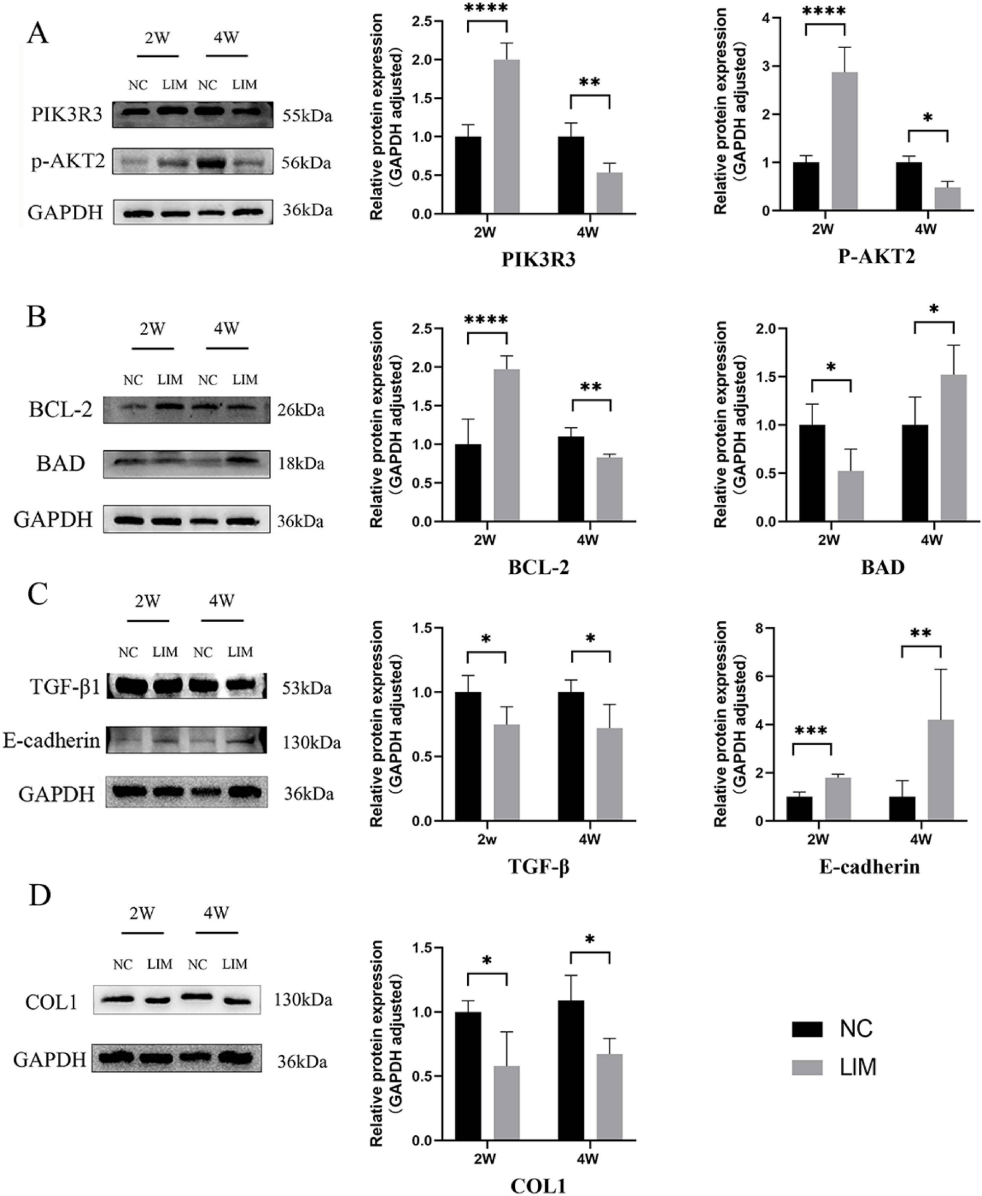
The relevant protein expression measured by western blot analysis. After 2- and 4-week myopia induction, expression of PIK3R3 and p-AKT2 (**A**), BAD and BCL-2 (**B**), TGF-β1 and E-cadherin (**C**), and COLI (**D**) in the sclera of the guinea pigs in the NC and LIM groups were detected by western blot analysis. Significance refers to comparisons of the LIM group to the NC group (* *p* < 0.05, ** *p* < 0.01, *** *p* < 0.001, **** *p* < 0.0001). *N* = 4.

As shown in Fig. 9, we noted that after 2 weeks of myopia induction, the levels of PIK3R3, and p-AKT2 in the sclera in the LIM group were significantly higher than the level of the NC group; however, after 4 weeks of myopia induction, the levels of PIK3R3 and p-AKT2 in the sclera in the LIM group decreased significantly compared with the level of the NC group (Fig. 9A, *P < 0.05, **P < 0.01, and ****P < 0.0001). Meanwhile, we also found that the level of the anti-apoptotic gene BCL-2 was up-regulated in the sclera in the LIM group after 2 weeks of myopia induction, whereas the pro-apoptotic gene BAD level was decreased. In contrast, the expression trend of the related molecules was reversed after 4 weeks of myopia induction (Fig. 9B, *P < 0.05, **P < 0.01, and ****P < 0.0001), suggesting that PIK3R3 and p-AKT2 may be correlated with cell survival and apoptosis. Nevertheless, we also noted that after myopia induction for 2 and 4 weeks, the expression of E-cadherin in the sclera in the LIM group was significantly higher than the level of the NC group, whereas the expression of TGF-β1 and COLI was continuously decreased (Fig. 9C, D, *P < 0.05 and **P < 0.01), indicating that the continuously elevated E-cadherin. The decreased levels of TGF-β1 and COLI were correlated with scleral remodeling. Importantly, continuous expression of these molecules was correlated with persistence of scleral remodeling.

### MMP2 and TIMP2 expression detected by ELISA

**Fig. 10:**
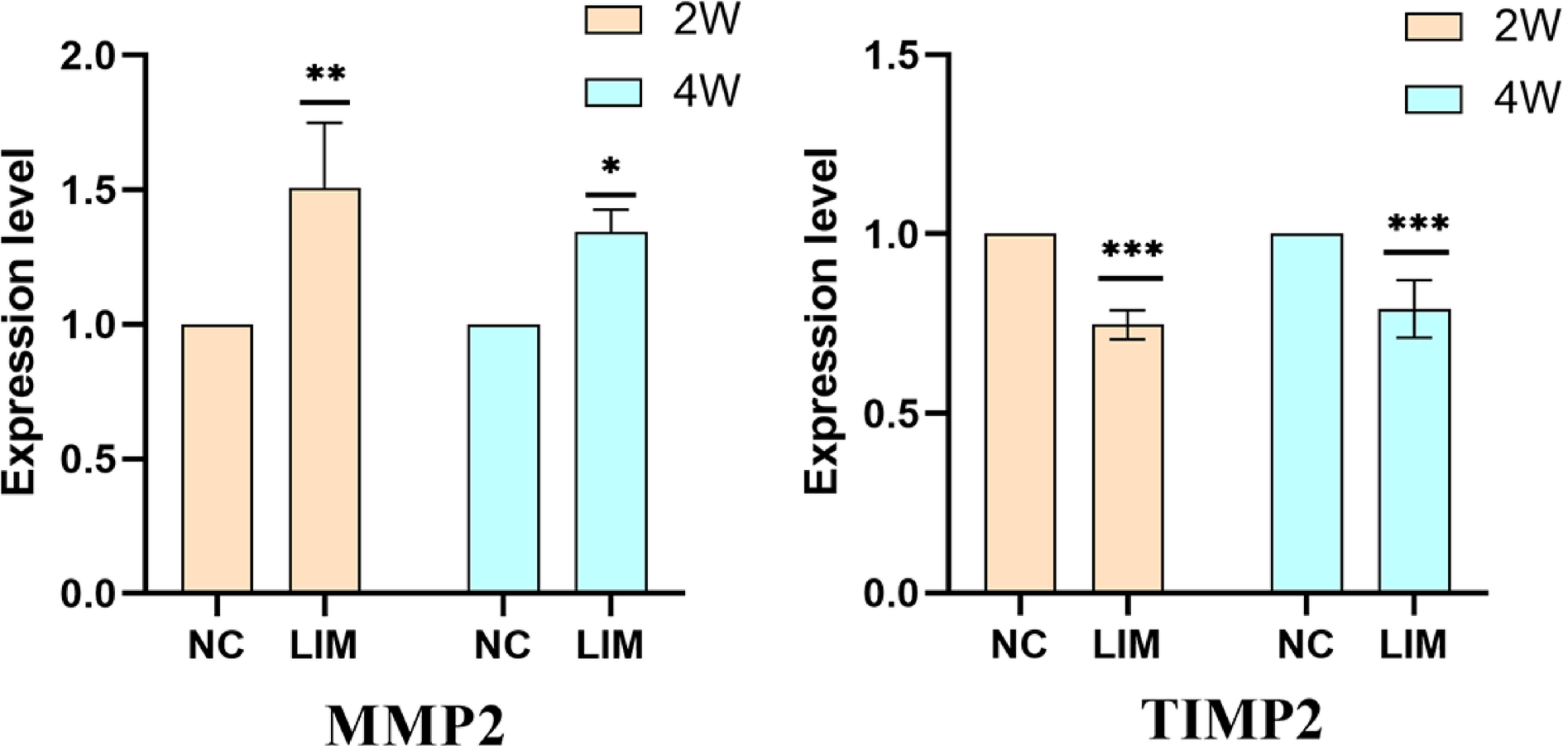
Relevant expression of MMP2 and TIMP2 in sclera tissues in the NC and LIM groups. The MMP2 and TIMP2 levels were detected by ELISA after 2- and 4-week myopia induction. Significance refers to comparisons of the LIM group to the NC group (* *p* < 0.05, ** *p* < 0.01, *** *p* < 0.001). *N* = 3.

After 2- and 4-week myopia induction, the MMP2 and TIMP2 expression in scleral tissues in the right eyes from the NC and LIM groups were detected by ELISA. The results showed that the MMP2 expression in the sclera of guinea pigs in the LIM group was increased compared with the levels of the NC group after 2 and 4 weeks of myopia induction. In contrast, the expression of TIMP2 in the LIM group was decreased (Fig. 10, *P < 0.05, **P < 0.01, and ***P < 0.001).

## Discussion

In this study, monocular application of -6.00D lenses was used to establish a guinea pig model of experimental myopia. After 2 and 4 weeks of myopia induction, we found that the refraction and axial length of LIM guinea pigs were significantly increased compared with those of NC animals, the refractive state became more myopic with the duration of lens wear, the sclera structure of myopic guinea pigs was disorganized and loose, and the number of collagen fibers was reduced. At the same time, we also noted that after 4-week myopic induction, the collagen fibers decreased, the muscle fiber cellulose increased and apoptotic cells significantly increased, indicating that scleral remodeling was involved in the occurrence and development of myopia by promoting the breakdown of collagen, cell transdifferentiation, and apoptosis.

PI3K plays a key role in many cellular processes, including glucose metabolism, apoptosis, cell proliferation, transcription, and cell migration. AKT is a serine/threonine kinase with three subtypes: AKT1, AKT2, and AKT3, and AKT kinase subtypes are generally thought to play overlapping roles in PI3K-mediated signaling. Notably, an increasing number of studies have confirmed that the PI3K/AKT signaling pathway can be abnormally activated in a variety of cancer^18–21^, pulmonary fibrosis^22,23^, Alzheimer’s disease, and Parkinson’s disease^24^. Thus, the abnormal activation of the PI3K/AKT signaling pathway is closely associated with the development of diseases. Based on these facts, we speculate that the disturbed PI3K/AKT signaling pathway may be involved in sclera remodeling in the development of myopia. Bao et al.^25^ demonstrated that the PI3K/AKT/ERK signaling pathway plays a critical role in retinal fibrosis during experimental myopia, showing that its activation promotes retinal ECM remodeling and fibrosis. In contrast, this study focuses on scleral remodeling and apoptosis in myopia progression, specifically examining the temporal dynamics of the PI3K/Akt signaling pathway. By shifting the focus from retinal fibrosis to scleral apoptosis, this study provides new insights into the molecular mechanisms driving myopia progression. Through IPA analysis, we found that the PI3K/AKT signaling pathway was significantly activated in the sclera of myopic guinea pigs, suggesting that the PI3K/AKT signaling pathway may be involved in myopia.

The differential expression of PIK3R3, AKT2, BCL-2, and BAD at weeks 2 and 4 reflects the dynamic changes in the PI3K/AKT signaling pathway in response to myopic stress. At 2 weeks, the upregulation of PIK3R3 and AKT2 indicates the initial activation of the PI3K/AKT pathway, promoting cell survival and inhibiting apoptosis. This is further supported by the increased expression of BCL-2, an anti-apoptotic protein, and the decreased expression of BAD, a pro-apoptotic protein. These changes suggest that during the early stages of myopia induction, scleral cells activate protective mechanisms to counteract stress and maintain tissue integrity. However, by 4 weeks, the expression of PIK3R3 and AKT2 significantly decreases, accompanied by a reduction in BCL-2 and an increase in BAD. This shift indicates the suppression of the PI3K/AKT signaling pathway, leading to a loss of cell survival signals and a subsequent increase in apoptosis. This transition reflects the sclera’s inability to sustain the protective response under prolonged stress, resulting in increased cellular apoptosis and tissue remodeling. The shift from cell survival to apoptosis aligns with the observed structural changes in the sclera, such as the disorganization of collagen fibers and thinning of scleral tissue, which are hallmarks of myopic progression. The TEM analysis showed that the morphology and structure of the sclera became thinner and disorganized after 4-week myopic induction; meanwhile, the result of Masson staining and immunohistochemical assay also displayed that the expression of collagen fibers in the sclera decreased in myopic guinea pigs, and TUNEL staining demonstrated that the number of cellular apoptosis increased. In addition, with the extension of myopic induction, the scleral morphology and structure changed. Based on these findings, we infer that the scleral remolding in myopic guinea pigs is involved in the changes in the scleral microenvironment. In other words, the alteration of the scleral microenvironment is involved in the changes of corresponding molecules under different stress.

Cellular stress is sensed by a few molecules and pathways that can participate in the regulation of cell survival or apoptosis, and the stress response depends on the degree of stimulation or the related molecule-mediated differential post-translational modifications^26^. Under stress, cells can regulate the expression of adhesion protein according to the change in pressure^27^. In the present study, we noted that under mild stress (stress lasting for 2-week myopia induction), activated PI3K/AKT signaling pathway promotes the expression of p-AKT2 and BCL-2, which can inhibit apoptotic signaling and contribute to cell survival. Conversely, activation of the PI3K/AKT signaling pathway will be inhibited under strong stress (stress lasting for 4-week myopia induction), which leads to the decreased expression of p-AKT2 and enhances BAD level, thereby initiating apoptotic signaling and causing apoptosis. A previous study showed that diabetic cardiomyopathy is usually accompanied by cardiomyocyte apoptosis, together with reduced AKT phosphorylation level, whereas curcumin treatment enhanced phosphorylation of AKT and attenuated apoptosis in the heart of diabetic rats^28^. In addition, Jeon and colleagues also revealed that overexpression of fragile X-linked mental retardation protein (FMRP) could activate the PI3K/Akt signaling pathway, enhance the expression of p-PI3K, p-AKT and Bcl-xL, thereby promoting the HeLa cell survival^29^. Similarly, our previous investigation has revealed that 2-week experimental myopic induction could activate the RASA1 signaling pathway, which contributes to cell survival, whereas 4-week myopic induction can inactivate the RASA1 signaling pathway, promoting cellular apoptosis of the choroidal tissues^30^. Liu et al. discovered lens-induced myopia can decrease the miR-92b-3p expression and enhance the BTG2 level, causing apoptosis of retinal cells^31^. Based on these findings, we hypothesize that the level of PIK3R3 was influenced by different stress, which can further either stimulate phosphorylated AKT or inhibit phosphorylated AKT, affecting cellular survival or apoptosis. Elevated expression of phosphorylated AKT under mild stress will enhance cellular survival, whereas the decreased level of phosphorylated AKT under strong stress will exaggerate cellular apoptosis. All these changes will influence the balance of the scleral microenvironment and, further affect scleral remodeling.

The sclera is a fibrous connective tissue consisting largely of heterologous collagen fibrils that comprise mainly typeI collagen with small amounts of other fibrillar and fibril-associated collagens. During the process of myopia, TGF-β can influence cell growth, differentiation, and migration in the exaggerated scleral growth^32^. TGF-β is a key mediator of collagen synthesis^33^, and its low expression will lead to reduced collagen synthesis and influence scleral remodeling^34^. E-cadherin is the main component of epithelial adherens junctions (AJs) that plays a fundamental role in the maintenance of stable cell-cell adhesion and overall tissue integrity^35^. E-cadherin protein (encoded by the CDH1 gene) is essential to epithelial polarization and differentiation, and environmental stimuli can affect its expression^36^. Scleral remodeling can be influenced by the overload pressure, the increased matrix degradation, and decreased collagen fibers, inducing the occurrence of increasing scleral extracellular matrix breakdown and resulting in the up-regulation of E-cadherin expression. Studies have shown that MMP2 can degrade collagen, proteoglycan, and other components of the scleral outer membrane^37^, thereby inhibiting fibrosis, while TIMP2 and MMP2 antagonize each other and have the opposite effect. Thus, the balance between MMP2 and TIMP2 is critical for normal scleral matrix turnover and subsequent regulation of eye development. In this study, we found that after 2 and 4 weeks of myopia induction, the expression of TGF-β1, COLI, and TIMP2 decreased compared with the levels of normal guinea pigs, whereas the expression of E-cadherin and MMP2 increased. In addition, E-cadherin was highly expressed in the scleral tissues in myopic guinea pigs, whereas COLI was significantly decreased. These findings further confirm that the changed microenvironment of the sclera will influence scleral remodeling during the development of myopia.

**Fig. 11:**
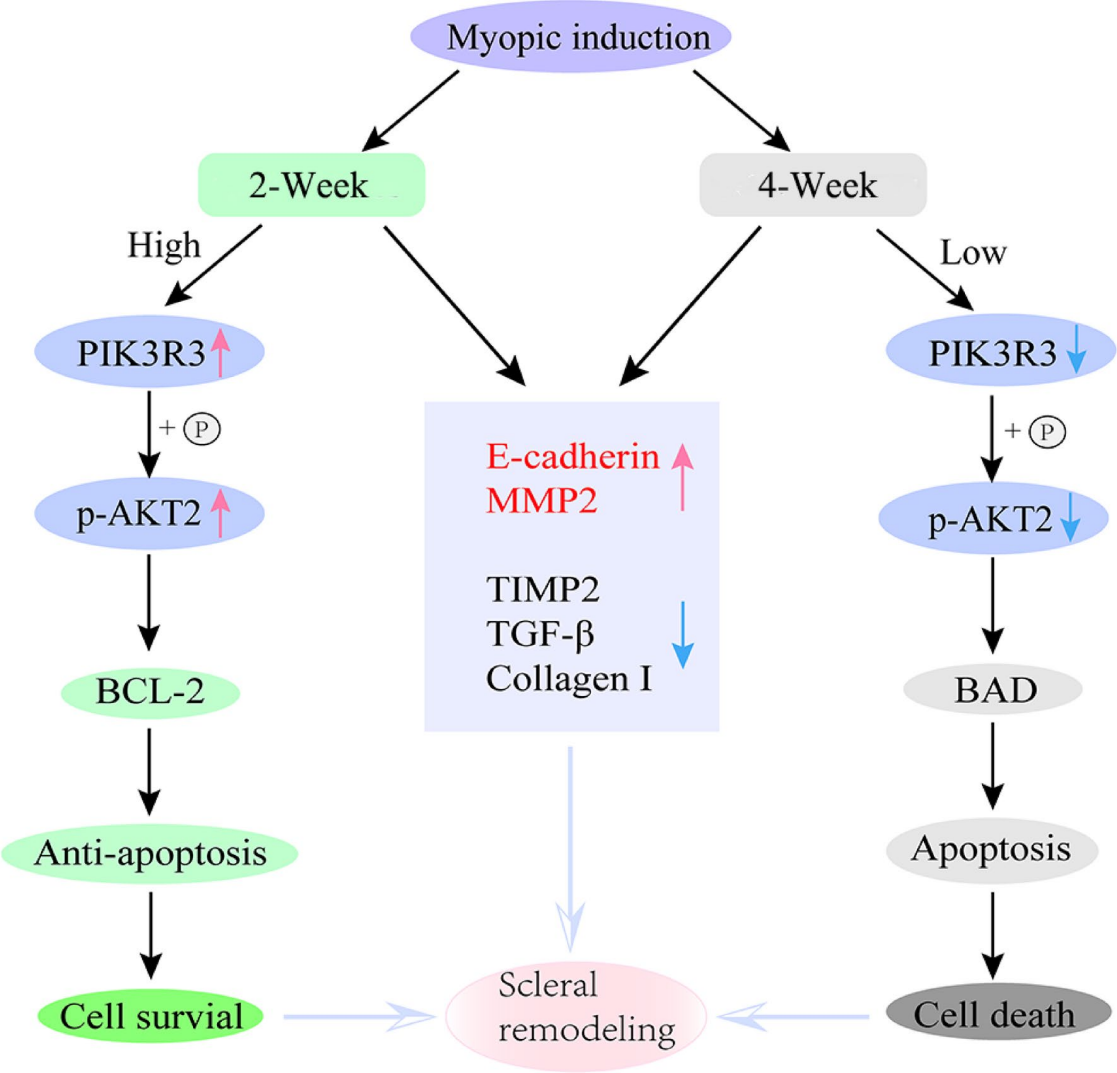
A schematic illustration of scleral remodeling in myopic guinea pigs via regulating the PI3K/Akt signaling and its related molecules.

As illustrated in Fig. 11, we presume that the activated PI3K/Akt signaling pathway would promote cell survival, whereas the inactivated PI3K/Akt signaling pathway would enhance cellular apoptosis. Both activation and inactivation of the PI3K/Akt signaling pathway regulate the related molecule expression in the process of myopic development and induce the change of the scleral microenvironment, thereby leading to scleral remodeling.

## Conclusions

In conclusion, this study confirmed that refraction and ocular length of experimental myopia guinea pigs gradually increased with the extension of myopic induction. The sclera of experimental myopic guinea pigs was thinner, the tissue structure was disorganized, the collagen fibers were reduced, and the apoptosis was increased, thereby leading to the alteration of the scleral microenvironment, and inducing sclera remodeling. Both activation and inactivation of the PI3K/AKT signaling pathway participate in the process of myopic development by regulating the related molecule expression. Our results gain new insights into scleral remodeling involved in the disturbed PI3K/Akt signaling is the cause of scleral remodeling via regulating molecules involved in collagen degradation and cellular apoptosis, providing ideas for the establishment of a new potential target in treating myopia.

## Electronic supplementary material

Below is the link to the electronic supplementary material.

Supplementary Material 1

Supplementary Material 2

## Data Availability

Data is provided within supplementary information files.

## Abbreviations

LIM: Lens-induced myopia
qPCR: Real-time quantitative PCR
ELISA: Enzyme-linked immunosorbent assay
MMP2: Matrix metallopeptidase 2
TGF-β1: Transforming growth factor-beta 1
TIMP2: Tissue inhibitor of matrix metalloproteinase-2
COLI: Collagen I
ECM: Extracellular matrix
PI3K/Akt: Phosphatidylinositol 3 kinase/protein kinase B
PIK3R3: Phosphoinositide-3-kinase regulatory subunit 3
PFA: Paraformaldehyde
DAPI: 4’6-diamidino-2-phenylindole
IPA: Ingenuity Pathway Analysis
PVDF: Polyvinylidene fluoride
BSA: Bovine serum albumin
SD: Standard deviation
FMRP: Fragile X-linked mental retardation protein
AJs: Adherens junctions
