# Supplementary figures and images for: Covariation of scleral remodeling and PI3K/Akt signaling pathway in experimental myopia
Source: Sci Rep. 2025 Apr 11;15:12476. doi: 10.1038/s41598-025-97643-7 (PMC11992133; doi:10.1038/s41598-025-97643-7)

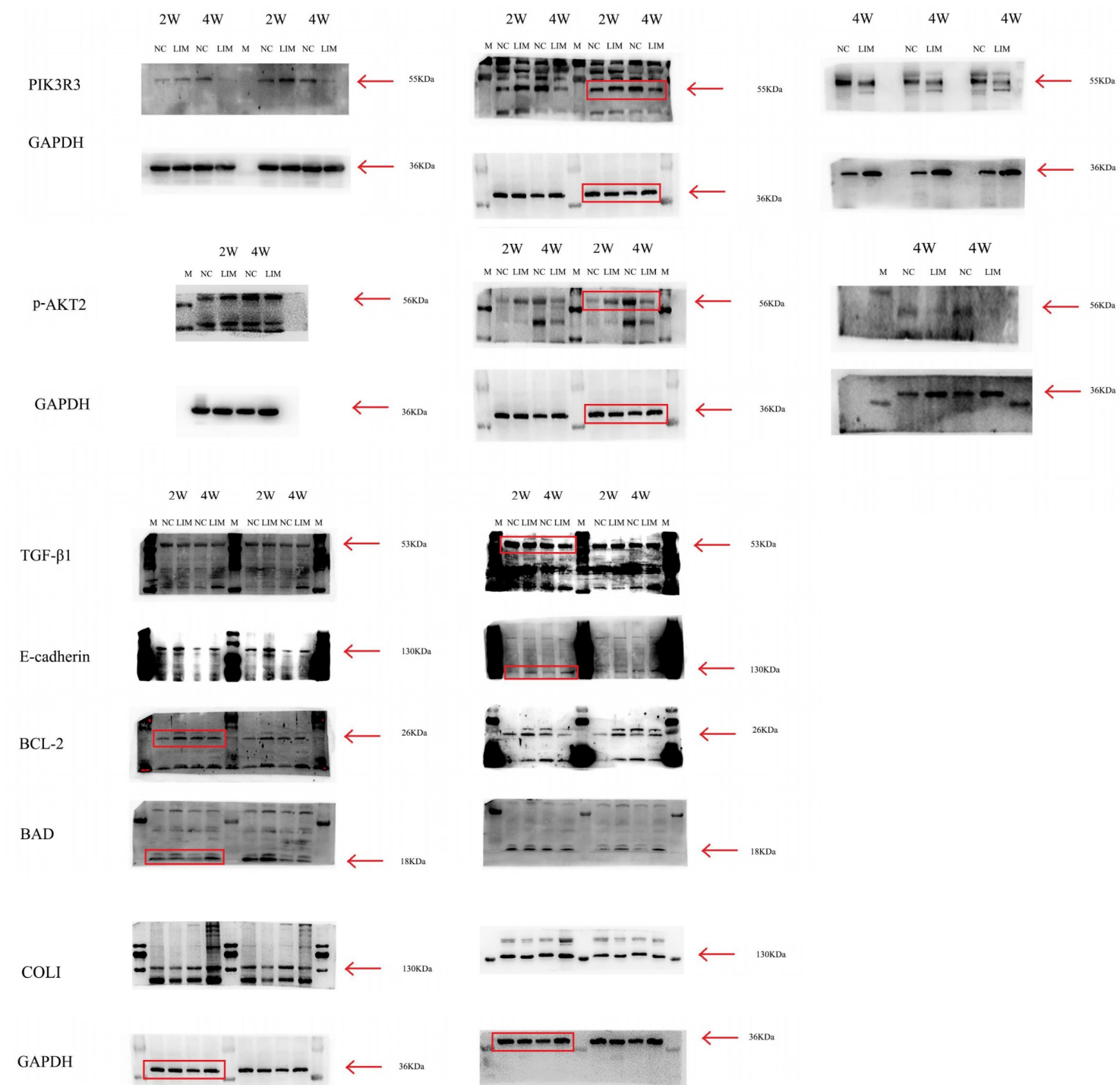

The target proteins in the red rectangle were shown in the paper.

Supplement: Supplementary file 2 — Supplementary Material 2 [file 41598_2025_97643_MOESM2_ESM.pdf]
